# Microbiome yarns: microbiome basis of memory^1^,^2^,^3^,^4^

**DOI:** 10.1111/1751-7915.12849

**Published:** 2017-10-02

**Authors:** Kenneth Timmis, Franziska Jebok, Gabriella Molinari, Manfred Rohde, James Kenneth Timmis

**Affiliations:** ^1^ Institute of Microbiology Technical University Braunschweig Braunschweig Germany; ^2^ Institute for Educational Science University of Freiburg Freiburg Germany; ^3^ Central Facility for Microscopy Helmholtz Centre for Infection Research Braunschweig Germany; ^4^ Department of Surgery and Cancer Imperial College London London UK

## Part 1

*GET (Global Environment Television), Studio 7A, BBZ Plaza, Burbank, 7.30 pm: Abigail Repor‐Tastory, Discovery Presenter, turns to face the camera*: Good evening and welcome to a new episode of ‘Discoveries that Change our Lives’. Our guest this evening is once again Dr. Anastasia Noitall‐Most^1^ from the Streber Elite University of Los Angeles. Good evening Dr. Noital‐Most *(shaking hands)* and thank you for appearing on the program.

*Dr. Noitall‐Most:* Good evening Abi; it is always a pleasure to be here.

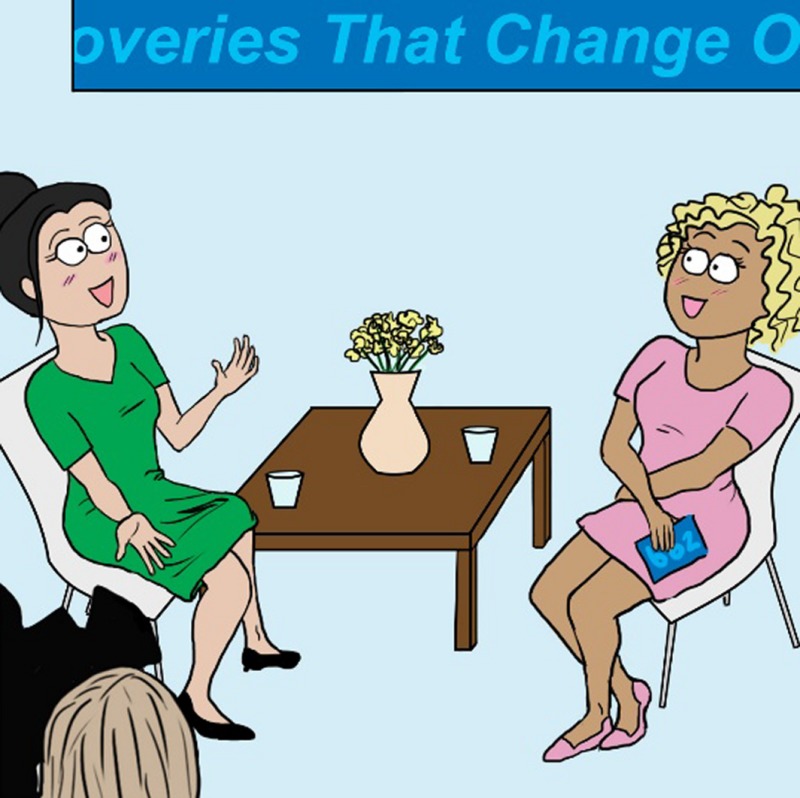

*Ms. Repor‐Tastory* Ani: this evening we want to discuss what seem to be amazing and game‐changing discoveries about our microbiome and memory.

*Dr. Noitall‐Most:* Exactly! As we know, a significant aspect of diminishing life quality in older citizens is memory loss – where did I put my keys/spectacles/pill box/walking cane/spare batteries for my hearing aid? what is that password/my grandchild's name? who is Rupert, in this fascinating lurid gossip? where did I park the car? what *is* the difference between caffè latte, cappuccino and macchiato? and so on. So there is an urgent need to find a means of slowing down or stopping memory loss. But, to do this, we need to know what memory really is. Until now, however, despite billions of dollars being invested in neurobiology, we still have not been able to figure out the mechanism responsible for memory and its age‐related deterioration. As one might expect, there are a number of theories based on correlations, including ageing‐related changes in neural matrix proteins, epigenetic processes, oxidative damage, and so on, but no defined mechanism. But now, several new, mostly serendipitous, discoveries have consigned all such theories to the bin.

*Ms. Repor‐Tastory:* This sounds really exciting; so what are the discoveries?

*Dr. Noitall‐Most:* Well: just a couple of introductory words to provide context. As you know, all our external body surfaces, including surfaces of the intestine, airways, etc., are covered with vibrant, thriving microbial communities that are not only nourished by our body secretions and the food we eat, but also contribute in a major way to our own body functions, such as digestion, vitamin acquisition, defences against infections, and so on. Since these microbial communities are a vital part of each and every one of us, we now consider them to be an integral part of an individual's identity – the human biome, which is the human plus its microbiome. Well, until now, the microbiome was always perceived as the ‘microbial skin’, an additional layer to our external surfaces, and the true interface between us and the environment.

But then an amazing discovery resulting from a rather grizzly happenstance radically changed this perception. On May 1^st^ last year, the bodies of 13 hikers were found in a forest clearing, deep in the Berg Mountains of Northern Germany. Apparently, the unfortunate people had died the previous night in somewhat suspicious circumstances.

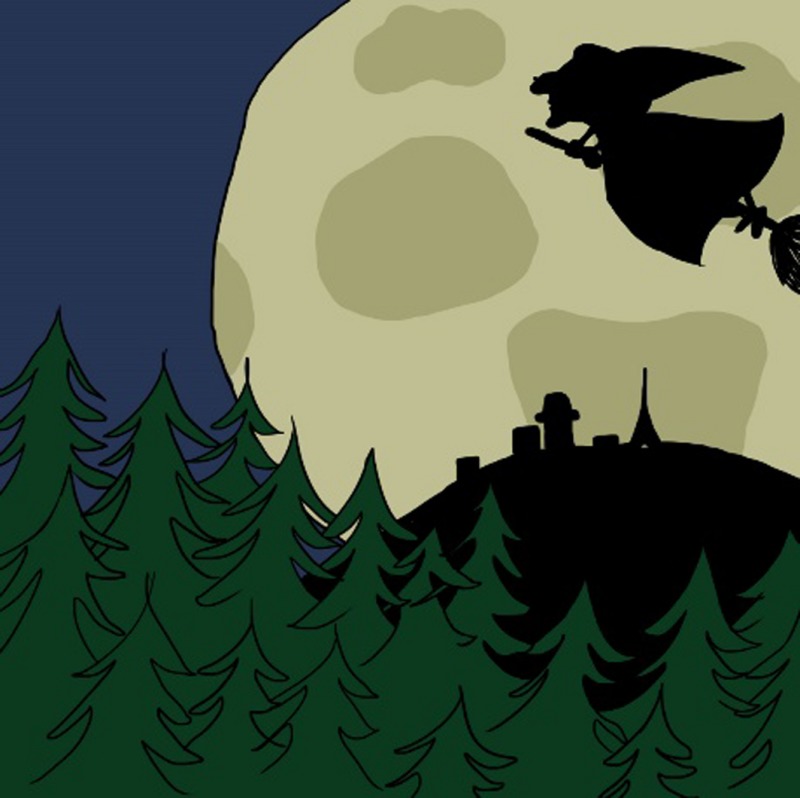

Anyway, since some of these poor souls had previously authorized the use of their body parts for transplantation and medical research, for some reason or other, after the post‐mortems, it was decided to examine their brain tissues. This led to an amazing discovery by the Imaging Group of Mabriella Golinari and Ranfredy Mohde of the Walpur Gisnacht Institute for Cellular Pathology in Bad Hurzbarg in Northern Germany.

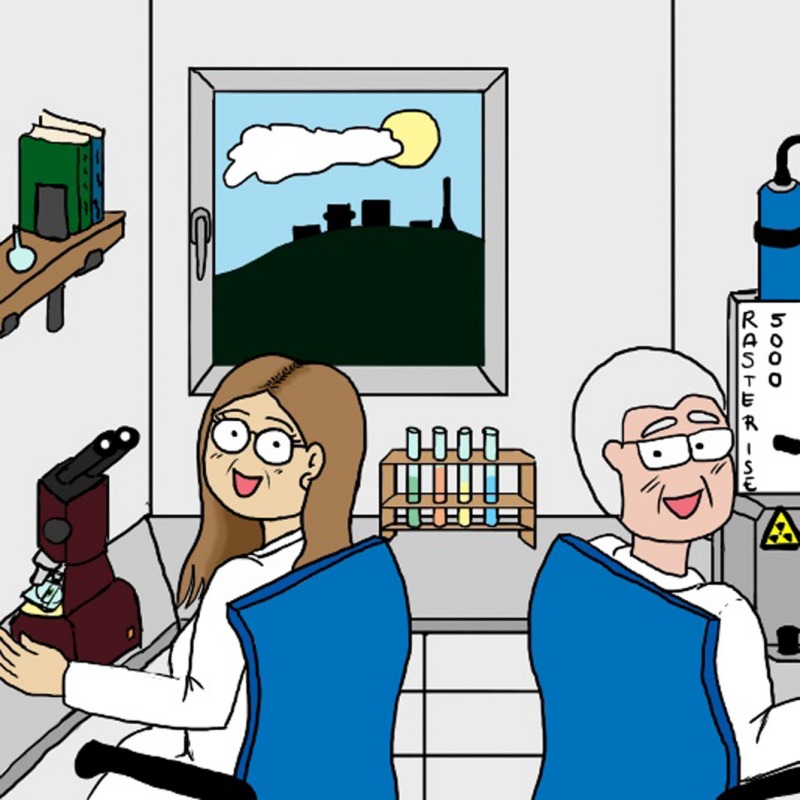

Using state‐of‐the‐art microscopic methods, they found exceptionally small microbes – so‐called ultramicrobacteria^2^ or UMB – inside the brain cells, the neurones.

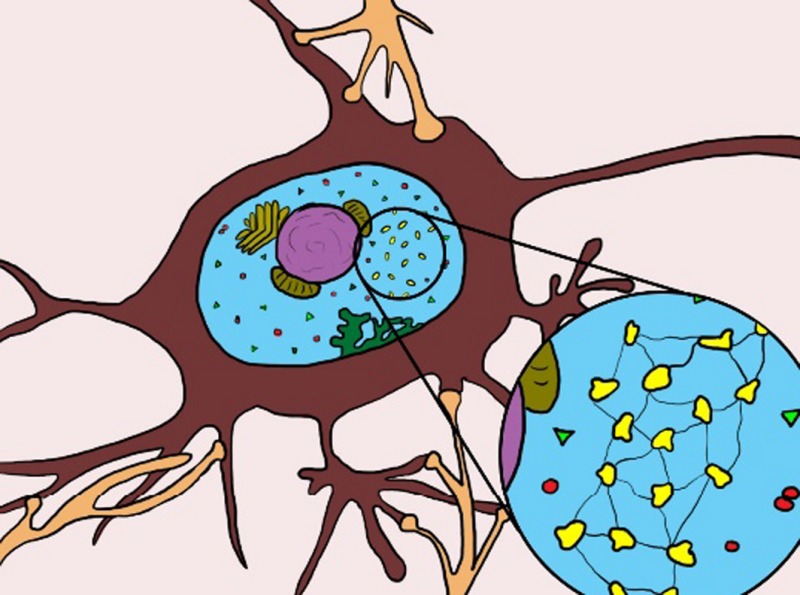

Well, of course, they then examined other organs of the deceased, but failed to find UMB in any other cells. They then obtained central nervous system samples from other cadavers and again found UMB in most samples. The very small size of UMB, and their presence only in neurones, explains why they had not been detected previously.

*Ms. Repor‐Tastory:* Wow, Ani, this is mind‐blowing! We have an unsuspected army of bugs living within our brains?

*Dr. Noitall‐Most:* Absolutely! So now we know that our microbiome not only consists of microbes that live on our body surfaces, but also of microbes living within our body cells.

Actually, with hindsight, this is not at all that strange. All our cells contain *mitochondria* – little intracellular organs, so‐called organelles – that produce energy for us from the food we eat. Mitochondria are actually a stripped‐down version of a bacterium that, in the mists of evolution, invaded a primordial cell, found it to be a rather cosy environment and decided to make itself useful. So, it made a pact with this early cell: in return for receiving its own food, it would convert all the remaining food to energy – it would become the power plant of the cell.^3^

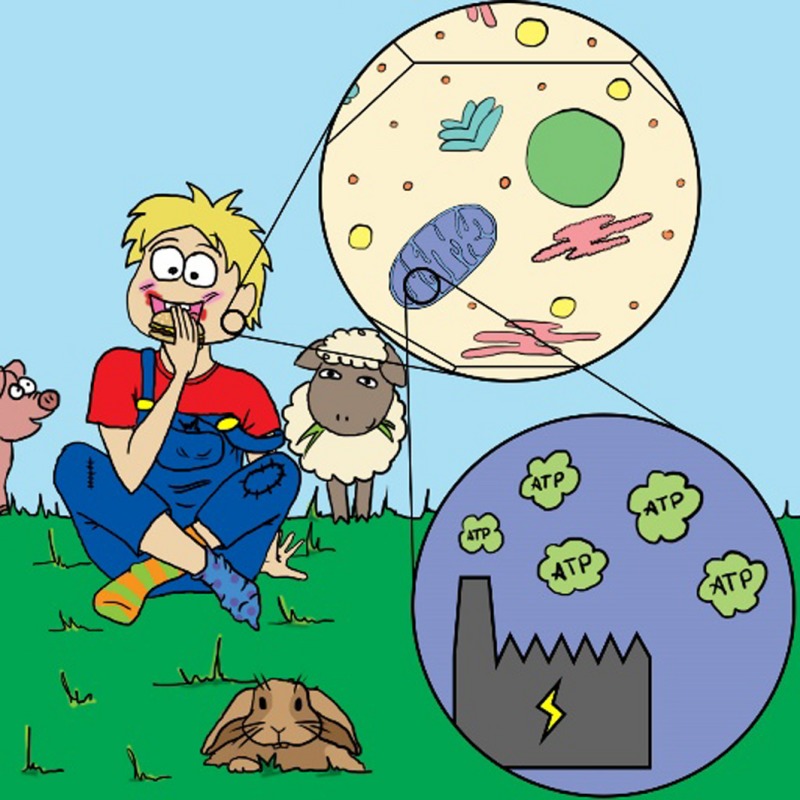

In other words, the bacterium entered into a symbiotic, or mutually‐beneficial, relationship with the cell to become a so‐called endosymbiont. During evolution, it progressively abandoned genes that were surplus to requirements and ultimately became simply a component of the cell. So the thing is: endosymbionts are not a new phenomenon for us. And we also know that different types of endosymbionts are regular inhabitants of diverse cells of many insects, plants, and other beings.^4^

*Ms. Repor‐Tastory:* Ani: this is absolutely fascinating! It seems that there is no end to new aspects of our microbiomes!

*Dr. Noitall‐Most:* Quite! The second discovery that revolutionised our view of memory was made by a mother‐daughter researcher pair – Professor Anna‐María Nesía and Dr. Rahel Anna‐María Nesía – of the Human Neurobiology and Microbiome Centre in Barcelona.

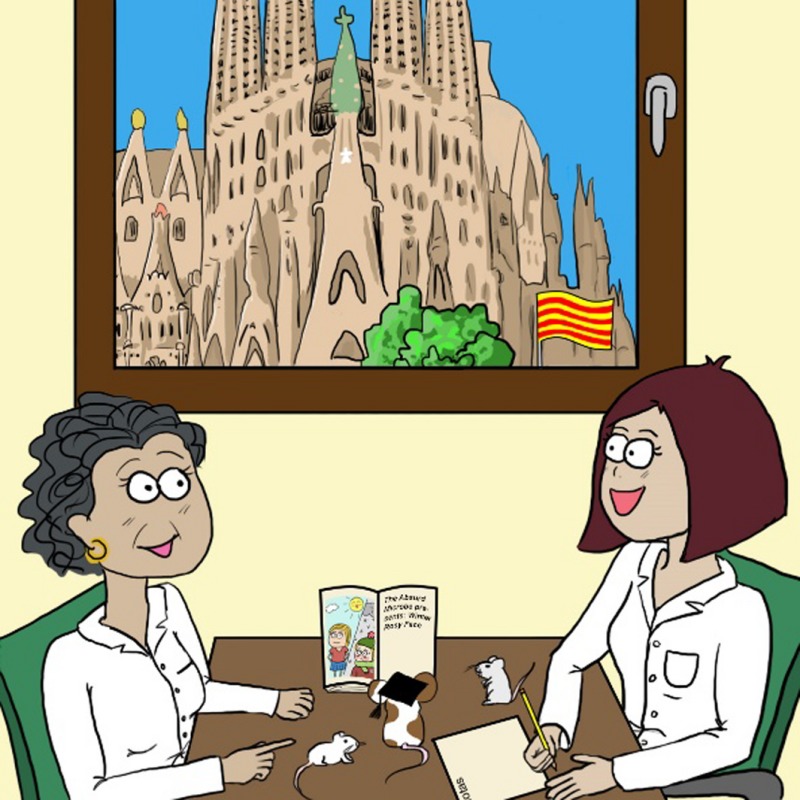

As soon as Professor A. M. Nesía learned of the UMB endosymbionts in neurones, she immediately went about trying to cultivate the bugs in the laboratory, in order to be able to investigate their activities. After much effort – it was not easy to find a growth medium equivalent to the inside of a neurone – she was successful and able to cultivate and study the bugs in detail and, in collaboration with the German microscopists, found that UMB have two important properties. The first is that they carry out the process of biomineralisation; that is: as part of their normal metabolism, they deposit minerals.^5^

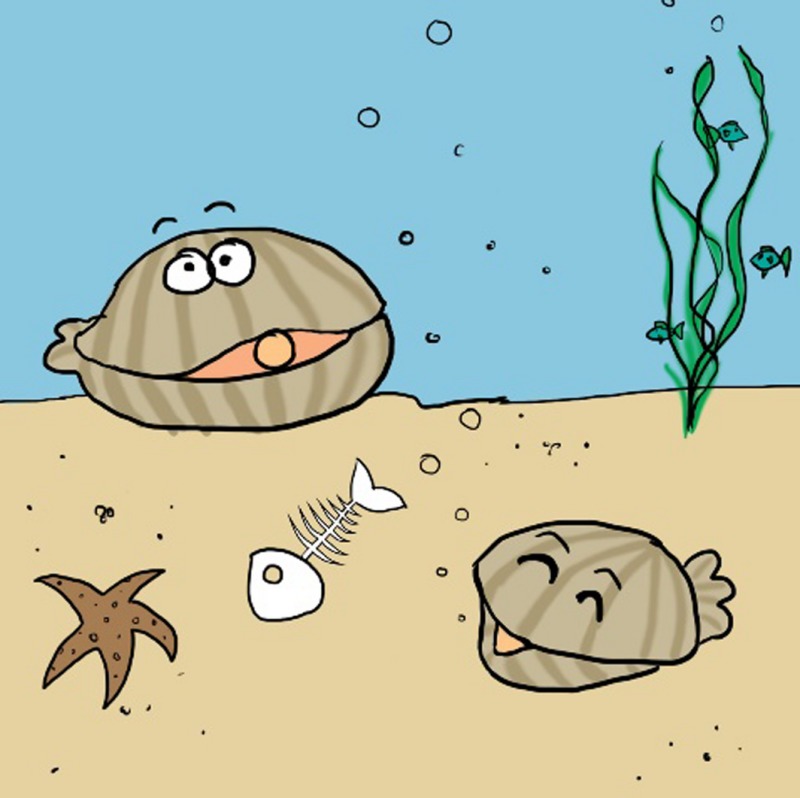

Of course, biomineralisation is a well‐known process in biology, as exemplified by sea shells made by molluscs, and bones made by vertebrates, like us, and many organisms and microbes create and deposit minerals outside or inside their cells. But the minerals deposited by UMB endosymbionts were found to be special: they are nanoparticles^6^ of silicon, called Si‐NPs for short.

*Ms. Repor‐Tastory, scratching her head and interjecting:* Wow, Ani… so UMBs are producing mini sand grains inside our brains – no wonder my head itches all the time!

*Dr. Noitall‐Most:* No, Abi: that is most certainly due to the shampoo you use. But back to the story: the second property of the cultivated UMB is that they produce long so‐called bacterial nanowires – filamentous extensions of the bacterial membrane that conduct electricity.^7^ These have been seen before in certain soil bacteria and their function is to acquire, channel and manage energy under conditions of oxygen deprivation. In fact, nanowires are currently at the centre of the booming business of bioelectricity production and the recycling of wastes by means of fuel cells^7^. Well, these findings led the two teams to examine neurones from fresh cadavers, and what they saw was absolutely stunning: the UMB contained Si‐NP arrays embedded in a regular lattice of so‐called redox proteins and iron‐sulfur clusters, and connected by nanowires,^8^ in a kind of wiring macramé.

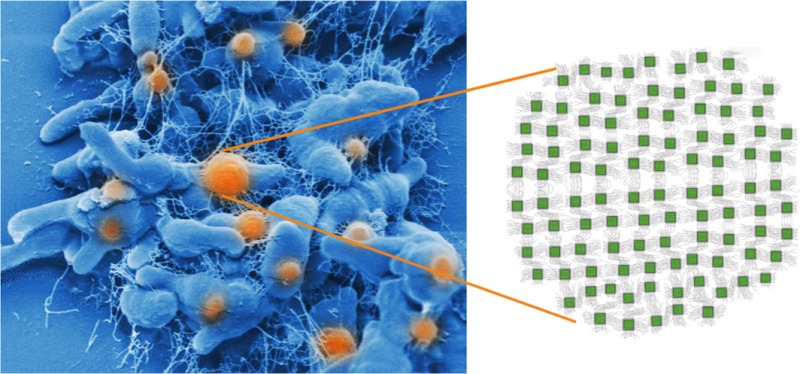

Now, as you know, silicon oxides have semiconducting properties and are used extensively in the manufacture of computer memory chips. Indeed, the images of Si‐NPs taken by the German group resemble microscopic computer memory chips. Moreover, the UMB not only had internal nanowires connecting the Si‐NPs with one another, and with the cell membrane, but also external nanowires linking UMBs together, and running inside the length of the neurone and connecting the Si‐NPs of its UMBs with those of other neurones. In other words, the entire central nervous system has a sort of microbial wiring replica that resides within it. So the big question was: what is the microbial replica doing, and are some of the roles we previously attributed to neurones actually being carried out by the UMB?

*Ms. Repor‐Tastory:* Amazing! Well: on that note, we'll take a break and return to this fascinating story in a few minutes to find out how endosymbionts are the basis of memory loss in the aged.

## Part 2

*Ms. Repor‐Tastory:* Welcome back viewers! Tonight, we are exploring with Dr. Noitall‐Most the basis of ageing‐related loss of memory. Ani: we have learned that our brains are full of bacteria producing things like computer chips: are our brains in reality just computers?

*Dr. Noitall‐Most:* Well, Abi: to investigate this further, Dr. R. A. M. Nesía created mouse mutants with altered neurone‐UMB relationships. And this approach brought even more weird and wonderful results. Among the rather comprehensive collection of mutant mice, one sat most of the day on its haunches, ignoring everything going on around it. It had a larger head than normal and was obviously very brainy, since it remembered all the learning exercises in record time. It was given the name Uni‐Don. Another was the opposite: it never remembered anything and when put in the treadmill, tried to run backwards for as long as it took to fall off exhausted. This mutant was called Sweaty Betty. Another seemed to have very selective memory since it remembered how to navigate a maze but not how to mate. This one was given the name Tristram. Unfortunately, no offspring were forthcoming from this mutant, so it was not possible to study it long‐term, although some information was obtained by examination of its neurones.

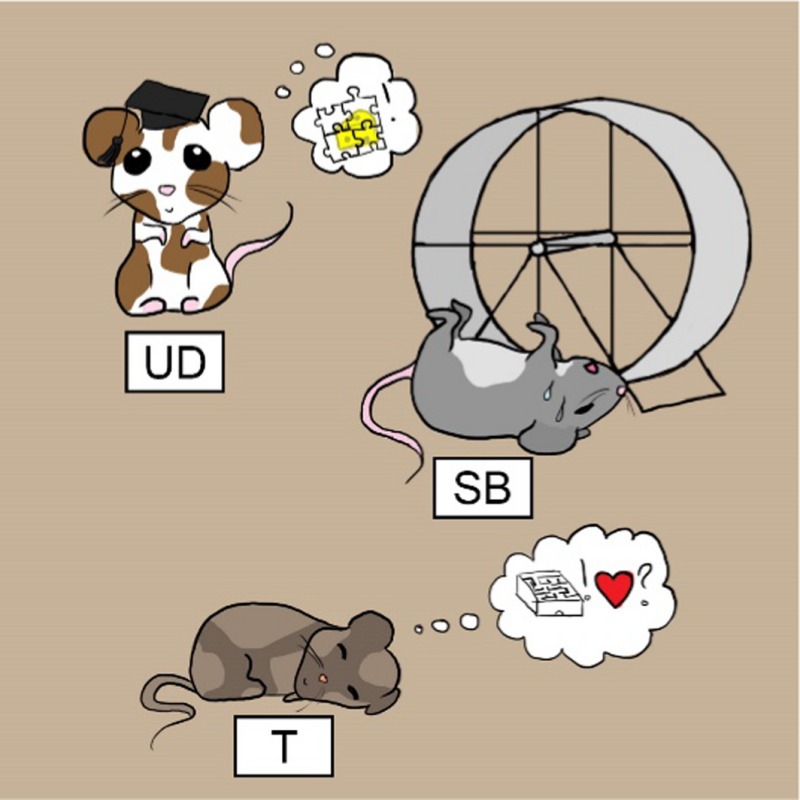

While all these strange phenotypes were fascinating, the most important mutant mice were those with neurones either lacking UMB or containing UMB that lacked Si‐NPs. In all cases, an absence of Si‐NPs was correlated with the complete absence of learning ability and memory. The crucial experiment was then to create a so‐called complemented mutant, a transgenic mouse mutant in which the normal UMB/Si‐NP content had been restored. In this case, memory was simultaneously restored, demonstrating causality between Si‐NPs and memory.

These experiments led the Nesías to hypothesise that the Si‐NPs are our brain's memory chips, that they receive information perceived by our sensory organs via the nanowires, that they organise and store the data, and that they enable it to be accessed and retrieved by the brain during the process of recollection. They named the UMB endosymbionts *Cognitianus siliconensis,* or CSIC for short.

*Ms. Repor‐Tastory:* Gosh, so we are nothing more than computer‐controlled robots!?

*Dr. Noitall‐Most:* Well, that is a rather dramatic representation of this information. What followed immediately after the formulation of the hypothesis was a large scale quantitative study of CSIC in brain tissues from human cadavers by Golinari and Mohde. What they found was that neurones from older people contained fewer Si‐NPs than those from younger people. This led to the logical hypothesis that age‐related memory loss is due to age‐related Si‐NP loss.

*Ms. Repor‐Tastory:* Wow! So is there any evidence for the proposal that memory deterioration in seniors is really due to loss of Si‐NPs?

*Dr. Noitall‐Most:* Yes, Abi, there some interesting new results. As we know, age‐related memory loss is selective – short‐term memory deteriorates more rapidly than long‐term memory.

*Ms. Repor‐Tastory, excitedly:* Yes, Ani: I rapidly forget what passwords I created just a day or so earlier, but can remember like it was yesterday all the glorious details of my rather athletic first date 15 years ago with my personal trainer at the fitness club*… (angry noises emanating from the in‐ear headphone)*… Oh, sorry, I did not mean to interrupt…

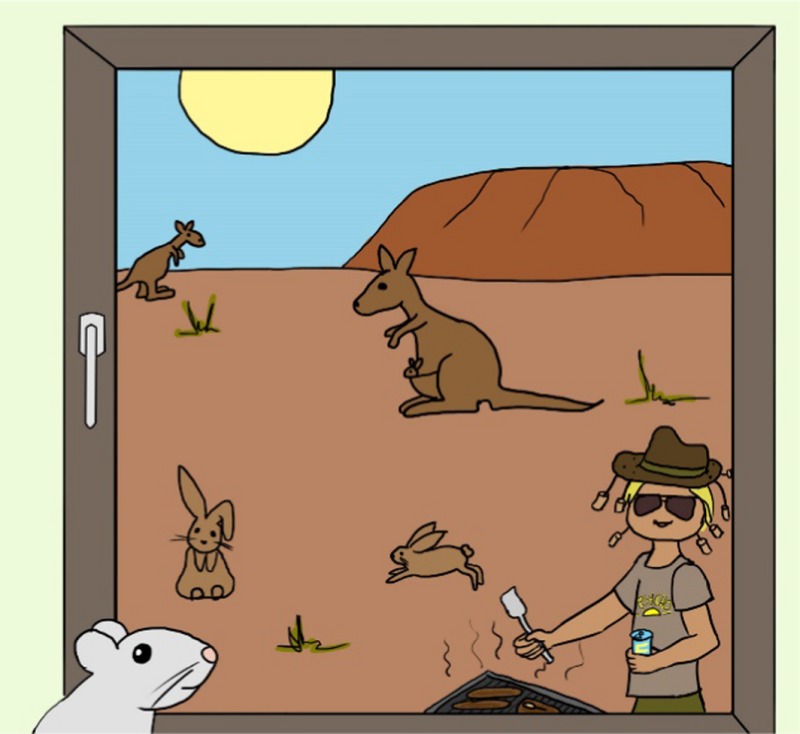

*Dr. Noitall‐Most, wistfully:* Yes, I know what you mean…. Anyway, to obtain insight into the basis of short‐ and long‐term memory, the Nesías decided to recruit the help of two behavioural scientists, Drs. Mel Bawn and Joe Lee Swagman, at the Australian National Institute of Marsupial and Rodent Psychology in Alice Springs.

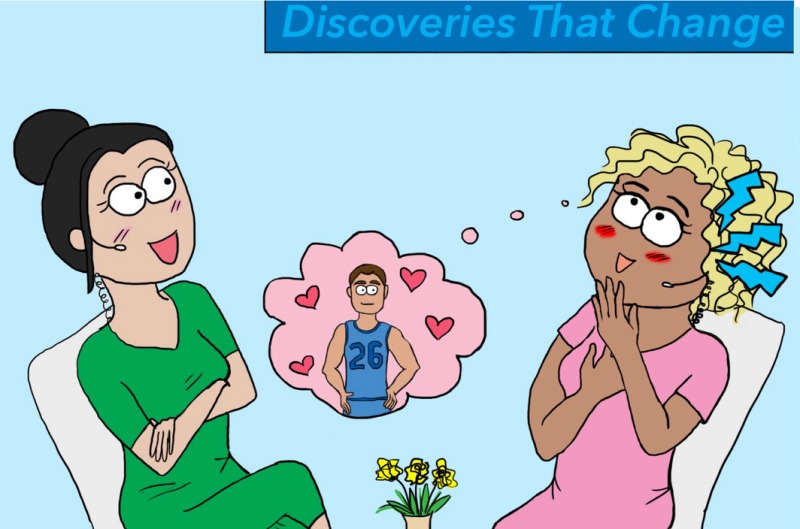

Mel and Joe took two groups of normal adult mice, having human age equivalents of 20 and 65, respectively, and taught them to lie on their backs and wiggle their tails in anticipation of a piece of cheese. Members of the two groups were then tested after 2 days or 20 days for their ability to repeat the exercise in response to the cheese cue. What this experiment showed was that older mice had a lower count of Si‐NPs in their CSIC cells and performed badly with regard to short term memory, though not long‐term memory. Younger mice performed equally well after 2 and 20 days. Interestingly, the few mice that were old but nevertheless had good short‐term memory also had normal Si‐NP counts. Moreover, both young and old Uni‐Don mutant mice, though only willing to wiggle their tails without lying on their backs, performed equally well at both time intervals. This experiment showed that, in mice at least, Si‐NP levels correlate with short‐term memory. The researchers named the UMB from the mouse, which is related to, but not identical with CSIC, *Cognitianus siliconensis, strain rodentis* or CSIRO for short.

*Ms. Repor‐Tastory*: Well, this is so exciting! So now we know the basis of memory failure in seniors.

*Dr. Noitall‐Most:* Yes, Abi: but there is still one major conundrum of memory to resolve, namely its assumed limited capacity, and the apparent unlimited capacity for sensory experience. To illustrate what I mean, consider the following: if you were lucky enough to obtain a table reservation at Frédy Girardet's^9^, or Ferran Adrià's^9^, your sensory organs would experience simultaneously the dynamics of continuously changing, complex, exquisitely subtle flavours and odours enticing and seducing your tastebuds and olfactory sensors, the diverse changing textures experienced by the lining of your buccal cavity, compounded by the visual sensual beauty of the ephemeral works of art they created on your plates, all accentuated and refined by an exceptional Richebourg created by Noblet (André/Bernard)^9^ or Pingus, and reinforced by feel‐good social chatter. Or imagine the sensations as you step outside at first light in late spring: the incredible smells, the dawn chorus of the birds, the sights of animals waking up and on the move hunting for food, the beauty of the cobwebs, the galaxy of stars created by light falling on the dew, the downy feel of the catkins…

*Ms. Repor‐Tastory, smiling wickedly:* … the sting of the sleepy wasp in a catkin, the tickling sensation of the mucous membranes and conjunctiva prior to the storm of histamine‐fuelled rhinitis and conjunctivitis….

*Dr. Noitall‐Most:* Hrmph, thank you Abi – very droll! Anyway, the fact that we are able to acquire and appreciate such multitudes of sensory experiences simultaneously testifies to our exceptionally high sensory capacity. However, it seemed unlikely that these enormous volumes of data could be stored for future recall on the limited data storage system we call memory.

*Ms. Repor‐Tastory:* Oh, right! Just think about the volume of routine data we continuously absorb throughout the day from our smart phones, computers, television, and so forth, much of which can be recalled, so is filed in memory. And imagine: all of this is amplified in the enhanced sensory states stimulated by recreational drugs. I remember being at a rock concert in San Diego a few years ago stoned out of^10^ …* (angry noises emanating from the in‐ear headphone)*… oh, sorry, I digress…

*Dr. Noitall‐Most:* Yes… well … in order to gain more insight into the issue of memory needs and available capacity, the Nesías consulted the renowned mathematician Professor Fidget Jones^1^, who modelled the various potential memory capacities on the basis of different assumptions about the Si‐NP functionalities.^11^

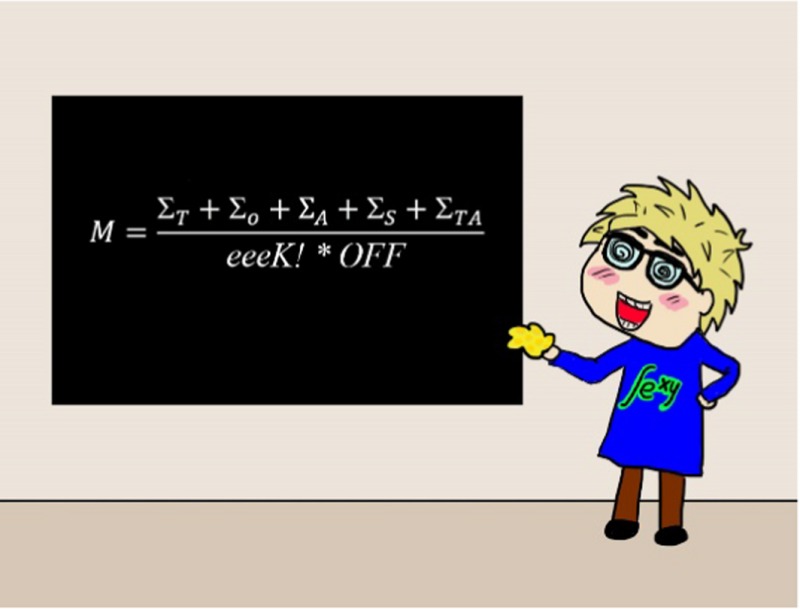

His findings were that (a) no plausible assumption would allow for storage of complete sensory information, that is, sensory information at the resolution originally acquired, and (b) even if memory consisted of relatively low resolution information, the capacity of the brain would still not suffice. He therefore concluded that (a) memory consists of low resolution information, for example, visual information is acquired and perceived in high definition and colour, but stored only at low definition in black and white, and (b) there must be an additional source of memory – an *external hard disk*, if you will.

Of course, when we remember, we *seem* to remember in high res and in colour, but this is almost certainly due to a sort of memory conditioning, for example, some sort of inate image augmentation of low res visual memory.

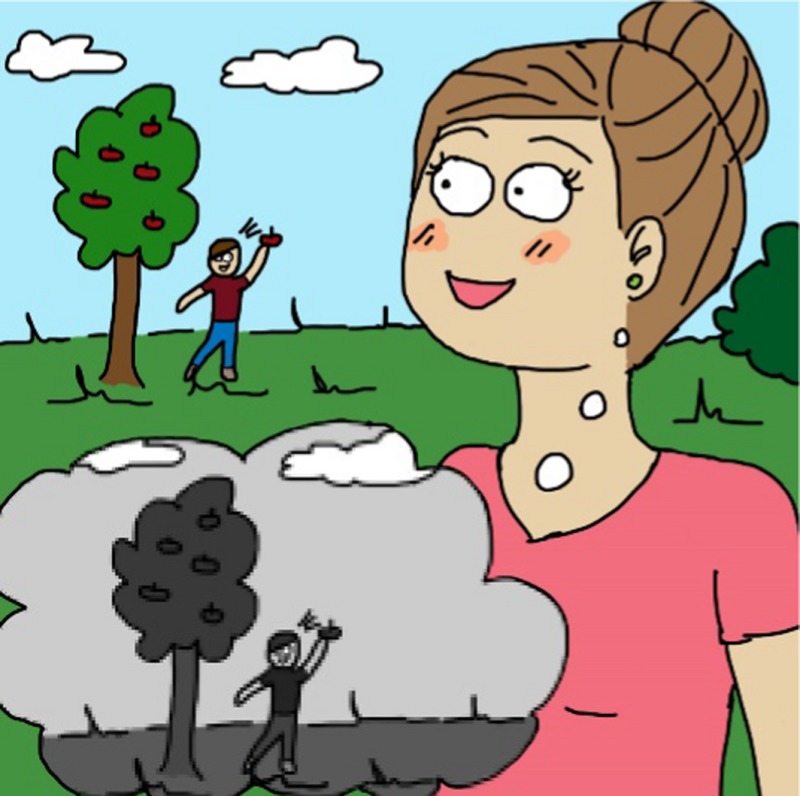

*Ms. Repor‐Tastory:* The need for an additional hard drive… gosh, we are just computers!?!

*Dr. Noitall‐Most:* Maybe not! But what could function as that additional hard disk? This question led the Nesías to our *second brain*. As you know, in addition to our central nervous system, there exists a second system of neurones associated with our gastro‐intestinal tract, known as the enteric nervous system, or ENS, that is connected with the CNS. Together, the ENS and the CNS form the gut:brain axis.

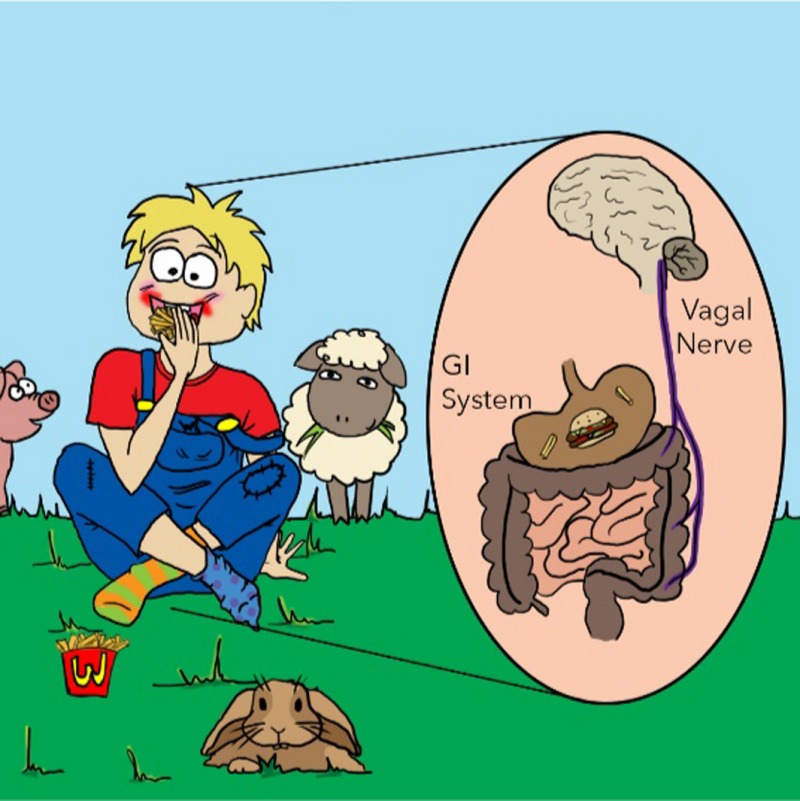

*Ms. Repor‐Tastory:* Yes: ‘butterfly stomach’ on a first date, gut feelings about share price upside, and stress‐induced comfort food‐booze bingeing, are all examples of communication between the gut and the brain.

*Dr. Noitall‐Most:* Quite so. And we have known for some time that the microbes in the GI tract communicate with the ENS and CNS, and thereby influence brain function^12^. At the moment, there is some very exciting research on gut microbiome‐brain interactions influencing the symptomology of conditions like autism, depression and some stress‐related disorders, and the possibility of treating them through gut microbiome manipulation is an incredibly exciting possibility that is being actively explored.^13^

Anyway, as expected, inspection of ENS neurones by Drs. Golinari and Mohde confirmed that they also contain UMB endosymbionts, and so the ENS probably functions as the second hard disk. And, importantly, the age‐related loss of UMB from ENS neurones is more marked than the loss from CNS neurones, suggesting that the ENS hard disk preferentially stores new information, and hence is responsible for short‐term memory.

So the current picture of memory is a sort of integrated database of low resolution information stored in Si‐NPs fabricated in the CSIC endosymbionts of our CNS and ENS neurones, and recallable via the nanowires. Our brain preferentially stores early and mid‐life memories, whereas the ENS preferentially stores late life memories, which it rapidly loses due to the high rate of attrition of CSIC in the ENS.

*Ms. Repor‐Tastory:* Incredible! And on that note, we'll take a break and return in a few minutes to complete this fascinating story

## Part 3

*Ms. Repor‐Tastory:* Welcome back viewers! Tonight, we are exploring with Dr. Noitall‐Most the basis of ageing‐related loss of memory. Now, Ani: we know what memory is and how it is lost; is there anything we can do to prevent or reduce loss of short‐term memory?

*Dr. Noitall‐Most:* This is of course the 64 billion dollar question, Abi. One interesting approach being taken at the moment is to see if we can gain useful insights from animals with exceptional memories – you will remember the adage ‘elephants never forget!’. Well, apparently, dolphins have even better memories.^14^ Latest research by the German and Australian groups on neurones from elephants and dolphins have revealed unusually dense clusters of Si‐NPs and nanowires in the neurones but, surprisingly, no UMB. The Nesías then showed that the key genes of the UMB have become integrated into the animal genomes, the endosymbionts have been lost, and high level Si‐NP production in neurones – but not in other cell types – takes place, thereby creating a higher memory capacity. Moreover, age‐related loss of Si‐NP is lower in such animals, presumably due to more efficient repair and/or replacement of damaged Si‐NP. Researchers at the Lorenzo von Syntech High Security Institute for Artificial Life in Madrid are currently integrating Si‐NP production genes from UMB into mouse genomes to see if this produces a mouse with a Uni‐Don phenotype.

*Ms. Repor‐Tastory:* But, Ani: practical application of this in humans would involve genome tinkering and would surely face enormous ethical and regulatory hurdles?

*Dr. Noitall‐Most:* Yes, Abi, you are absolutely right. Another promising approach being explored is faecal transplants, since much of our short‐term memory seems to be localised in our second brain which may be more readily influenced by changes in the gut microbiota. The Nesías have already made faecal transplants from Uni‐Don donors to normal mouse recipients and shown that the latter rapidly develop good short‐term memory. Human trials are now underway involving members of families with a history of good short‐term memory in old age as donors.

And perhaps the most exciting development: pre‐mortem and post‐mortem studies of people with eidetic/photographic memories, i.e. people whose normal memory capacity is exceeded by a huge factor by the actual capacity represented by their CSIC endosymbionts, has shown that they additionally have free‐living CSIC, that is, symbiotic but not endosymbiotic UMB, in their intestines covering the intestinal epithelium and communicating with the CSIC of the second brain. These individuals thus have a huge capacity *second external hard disk*. It remains to be seen if the free‐living CSIC, when transplanted to normal people, will create the network of connections needed for successful memory augmentation. In any case, the scientists involved in this work are already using the term bioaugmentation.^15^ There is, however, an ethical problem: should such transplants be allowable for – quote – normal people – unquote – who may be eager to acquire exceptional memories, such as gamblers, politicians, comedians, scholars, pub quiz addicts, spin doctors, historians, lawyers, and others, whose memory capacities may significantly influence their success, in order to unfairly acquire a competitive advantage?

*Ms. Repor‐Tastory:* Well, that is incredibly interesting! But are there any downsides to these new developments?

*Dr. Noitall‐Most:* This is of course a very important question and is giving headaches to quite a few folk. Some have raised the question of whether, if memory is just a microbial computer chip, it may become possible to hack into and steal information, or even to delete or reprogram memory, and whether it may ultimately become possible to create programmed humans/humanoids – that is, the opposite of artificial intelligence‐based humanised robots – which is very scary. These possibilities would seem to offer a lot of potential for criminal activity.

*Ms. Repor‐Tastory:* Gosh, this is mind‐boggling! And it begs the question: if memory, and therefore all knowledge, is stored in our bacterial endosymbionts, does this mean that our use of knowledge may be subjected to a sort of microbial gatekeeping?

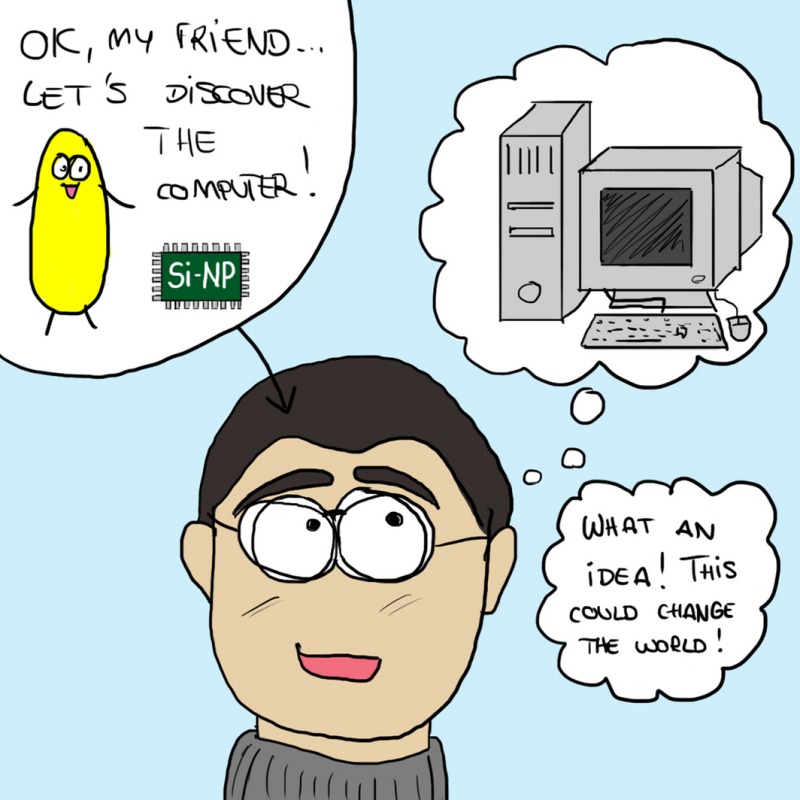

*Dr. Noitall‐Most:* Excellent point! Some people also wonder whether our anthropocentric view of the world, and our perception of self, will ultimately change in a significant way. They point out that microbes themselves have memory,^16^ that they co‐evolved with us and our ancestral forms, and consider it significant that we imagine we have discovered computing and silicon‐based memory, when the microbes had fabricated it for us from time immemorial. Given that gut microbes are involved in orchestration of brain development, function and behaviour,^9^ they wonder whether our microbes have instructed us to build computers based on their own design.

They even wonder whether the human is at the top of the evolutionary tree at all, or whether the microbes are; whether the human is just one of many animate scaffolds of microbial habitats, created to advance microbial interests, like the fungal gardens of ants; and whether our behaviour and activities are orchestrated for the greater microbial purpose. There exists the old phrase *mind over matter,* but now the phrase *microbial matter over mind* is being bandied about… But of course this is just idle speculation of the philosophers and ethicists…

*Ms. Repor‐Tastory:* Well – I am sure that these comments will feed the blogs for months. Thank you so much, Dr. Noital‐Most, for this fascinating exposé on memory and its progressive loss in the aging, and we look forward to having you again as guest on the program in future.

++++++++

## Conflict of interest

None declared.

## Funding Information

No funding information provided.

## Explanatory Notes for Online Version at www.theabsurdmicrobe.com

1 To view previous issues of *Microbiome Discoveries that Change our Lives*, go to www.theabsurdmicrobe/discoveries/oldstuff or see Timmis, K. and Timmis, J.K. (2017) Microbiomes, metabolic errors and detection of performance enhancing drug abuse. *Environm. Microbiol. Rep*. **9**: 25–26; Timmis, K., Jebok, F., Rigat, F. and Timmis, J.K. (2017) Microbiome yarns: microbiomology of curly and straight hair. *Microb. Biotech*. **10**: 231–237; Timmis, K. and Jebok, F. (2017) Microbiome yarns: microbiomology of winter rosy face. *Microb. Biotech*. **10**: 520–524, Timmis, K., Jebok, F. and Espejo, R.T. (2017) Microbiome yarns: microbial forensics for auditing provenance in global food chains. *Microb. Biotech*. **10**: 678–682.

2 Health Warning. This article is a figment of the authors’ imagination; any serious contemplation may unnecessarily challenge the mental harmony of the reader.

3 any possible resemblance of the fictional characters and organisations in this fable to a real person, organisation or enterprise is coincidental and unintentional.

4 This is dedicated to the memory of Daedalus, aka David Jones, the *court jester in the palace of science*, who invented Dreadco and, for 38 years, published in New Scientist and Nature all manner of fantasy discoveries and inventions, and amused generations of readers of these journals (e.g. https://www.washingtonpost.com/local/obituaries/david-jones-british-chemist-and-court-jester-in-the-palace-of-science-dies-at-79/2017/07/31/8dfcb0c4-75fb-11e7-9eac-d56bd5568db8_story.html?utm_term=.ee7355c18084), and to pioneering researchers of ultramicrobacteria, (endo)symbiosis, imaging, electro‐microbiology and bacterial nanowires, microbial biomineralisation, microbial nanoparticle generation, and, most particularly, of neuro‐micro‐biology research discovering microbiome:brain interactions that influence our behaviour, personalities and mental state. It is also dedicated to pioneering artists who explore our sensory capacities and, without prescription drugs, stimulate our pleasure centres to new highs, particularly Frédy Giradet, Ferran Adrià, Bernard Noblet, and others able to work miracles with simple ingredients gifted by the land.

5 youngest daughter of the renowned Dr. Noitall, see e.g. Koshland, D.E., Jr. (1994) Editorial: A simple proposal for health care rationing. *Science* **264**: 1515.

6 Kajander, E.O. and Çiftçioglu, N. (1998) Nanobacteria: an alternative mechanism for pathogenic intra‐ and extracellular calcification and stone formation. *Proc. Natl Acad. Sci. USA* **95**: 8274–8279; Luef, B., et al. (2015) Diverse uncultivated ultra‐small bacterial cells in groundwater. *Nature Communications*,; **6**: 6372 DOI: https://doi.org/10.1038/ncomms7372

7 Margulis L. (1970) *Origin of eukaryotic cells*. Yale University Press, New Haven, CT; Martin, W., Hoffmeister, M., Rotte, C., and Henze, K. (2001) An overview of endosymbiotic models for the origins of eukaryotes, their ATP‐producing organelles (mitochondria and hydrogenosomes), and their heterotrophic lifestyle. *Biol Chem* **382**: 1521–1539.

8 Stahl, D.A., Lane, D.J., Olsen, G.J. and Pace, N.R. (1984) Analysis of hydrothermal vent‐associated symbionts by ribosomal RNA sequence. *Science* **224**: 409–411; Stingl, U., Radek, R., Yang, H. and Brune, A. (2005) “Endomicrobia”: cytoplasmic symbionts of termite gut protozoa form a separate phylum of prokaryotes. *Appl Environ. Microbiol*. **71**:1473–9; Sironi, M., et al (1995) Molecular evidence for a close relative of the arthropod endosymbiont *Wolbachia* in a filarial worm. *Mol Biochem Parasitol*. **74**: 223–227.

9 Lin, W., et al. (2017) Origin of microbial biomineralization and magnetotaxis during the Archean. *Proc Natl Acad Sci U S A*. **114**: 2171–2176.

10 Macaskie, L.E., Empson, R.M., Cheetham, A.K., Grey, C.P. and Skarnulis, A.J. (1992) Uranium bioaccumulation by a *Citrobacter* sp. as a result of enzymically mediated growth of polycrystalline HUO_2_PO_4_. *Science* **257**: 782–784; Dillon, A., et al (2008) Metal oxide nano‐particles for improved electrochromic and lithium‐ion battery technologies. *Thin Solid Films* **516**: 794–797; Singh, O.V, Ed. (2015) Bio‐nanoparticles: Biosynthesis and Sustainable Biotechnological Implications. John Wiley and Sons;

11 Gemma, R., et al. (2005) Extracellular electron transfer via microbial nanowires. *Nature* **435**: 1098–1101; Gorby, Y.A., *et al*. (2006). Electrically conductive bacterial nanowires produced by *Shewanella oneidensis* strain MR‐1 and other microorganisms. *Proc. Natl Acad. Sci. USA* **103**: 11358–11363; El‐Naggar, M.Y., et al (2010) Electrical transport along bacterial nanowires from *Shewanella oneidensis* MR‐1 *Proc. Natl. Acad. Sci. USA* **107**: 18127–18131; Pirbadian, S. et al., (2014) *Shewanella oneidensis* MR‐1 nanowires are outer membrane and periplasmic extensions of the extracellular electron transport components *Proc. Natl. Acad. Sci. USA* **111**:12883–8; Sasaki S, Karube I. (1999) The development of microfabricated biocatalytic fuel cells. *Trends Biotechnol*. **17**:50–2; Rabaey K, Verstraete W. (2005) Microbial fuel cells: novel biotechnology for energy generation. *Trends Biotechnol*. **23**: 291–8; Nealson, K.H. and Rowe, A.R. (2016) Electromicrobiology: realities, grand challenges, goals and predictions. *Microb Biotechnol*. **9**: 595–600.

12 No, sorry: this is neither a nanobacterium, nor an endosymbiont, but anyway, so beautiful, that we decided to take full advantage of artistic licence and use it for illustrative purposes. Without decoration, it is designated 2D7, was isolated by Ken Timmis, Joan Timmis and Balbina Nogales from the phylosphere of some beautiful plant or other on the campus of the University of the Balearic Isles, while KT was a short‐term postdoc of Balbina Nogales, to whom he expresses infinite gratitude, and to the UIB for a grant supporting his stay in Palma. Back in Braunschweig, Gabriella Molinari and Manfred Rohde revealed the beautiful morphology of 2D7 and discovered that it is distantly related to *Quadrisphaera granulorum* (Maszenan, A.M., Tay, J.H., Schumann, P., Jiang, H.L. and Tay, S.T. (2005) *Quadrisphaera granulorum* gen. nov., sp. nov., a Gram‐positive polyphosphate‐accumulating coccus in tetrads or aggregates isolated from aerobic granules. *Int J Syst Evol Microbiol*. **55**: 1771–7.) which is, however, distinctly less winsome.

13
https://wine4soul.com/tag/fredy-girardet/; https://www.youtube.com/watch?v=Y4YNQnWkj7I; https://www.nytimes.com/interactive/2014/06/15/style/tmagazine/endofcuisine.html?_r=0; https://www.belgiumwinewatchers.com/en/journal/128/la-romanee-conti-the-grand-cru-of-burgundy

14 apologies for this gratuitous piece of artistic licence – urban legend suggests that memory of recreational drug‐enhanced experiences is not especially good

15 M = memory index, where M = 1 is plausible and M < 1 is implausible, and T = lifetime memorable (lm) tactile sensations experienced, approximated by the average number of times per day the smart phone is switched on x 94 x age; O = lm olfactory sensations experienced, approximated by the number of anti‐histamine tablets taken in the month of April x 100; A = lm auditory sensations experienced, approximated by the annual number of live concerts experienced x 1000 x 1/average decibel level; S = lm visual sensations experienced, approximated by the number of Instagram posts received during four consecutive weekends x 6573; TA = lm taste sensations experienced, approximated by the number of hamburgers eaten during the month of October x 803 (vegetarians were excluded from the modelling exercise); *Eeek!* = an uncertainty term that corrects for stochastic recollections of random events of the type “Eddie: I just remembered…I forgot to put some cod steaks back into the freezer after defrosting it the day before we caught the plane”, or “ Where are the children: I thought you had them? Surely we didn't leave them playing in the pub garden…?” and OFF = (no. of CSIC cells x the mean number of Si‐NPs per CSIC) x Z, a complexity factor encompassing Si‐NP half‐life, age‐related deterioration of sensory perception, and age‐ and diet‐related changes in intestinal microflora affecting ENS CSIC activities

16 https://www.ted.com/talks/heribert_watzke_the_brain_in_your_gut; Sampson, T.R. and Mazmanian S.K. (2015) Control of brain development, function, and behavior by the microbiome. *Cell Host Microbe*. **17**: 565–576; Dinan, T.G. and Cryan, J.F. (2017) Gut‐brain axis in 2016: Brain‐gut‐microbiota axis ‐ mood, metabolism and behaviour. *Nat Rev Gastroenterol Hepatol*. **14**: 69–70.

